# Impact of Tetrakis(dimethylamido)tin(IV) Degradation on Atomic Layer Deposition of Tin Oxide Films and Perovskite Solar Cells

**DOI:** 10.1002/smll.202404966

**Published:** 2024-11-06

**Authors:** Shuang Qiu, Augusto Amaro, Diana Fabulyak, Julien Appleby‐Millette, Cassidy Conover, Dongyang Zhang, Vishal Yeddu, I Teng Cheong, Irina Paci, Makhsud I. Saidaminov

**Affiliations:** ^1^ Department of Chemistry Department of Electrical and Computer Engineering Center for Advanced Materials and Related Technologies (CAMTEC) University of Victoria Victoria British Columbia V8P 5C2 Canada; ^2^ Seastar Chemicals ULC 2061 Henry Avenue West Sidney BC Canada V8L 5Z6 Canada

**Keywords:** atomic layer deposition, electron transporter layer, perovskite solar cells, tin oxide

## Abstract

Tin oxide (SnO_x_) films synthesized by atomic layer deposition (ALD) are widely explored in a range of optoelectronic devices including electrochemical sensors, transistors, and photovoltaics. However, the integrity of the key ALD‐SnO_x_ precursor, namely tetrakis(dimethylamido)tin (IV) (TDMASn), and its influence on the properties of ultimate films remain unexplored. Here a significant degradation of TDMASn into bis(dimethylamido)tin(II) via the Sn‐imine complex is reported, and its impact on the corresponding films and devices is examined. It is found, surprisingly, that this degradation does not affect the growth kinetics and morphology of ALD‐SnO_x_ films. But it notably deteriorates their electronic properties, resulting in films with twice the electrical resistance due to different oxidation mechanisms of the degradation products. Perovskite solar cells employing such films exhibit a significant loss in power conversion efficiency, primarily due to charge transport and transfer losses. These findings urge strategies to stabilize TDMASn, a critical precursor for ALD‐SnO_x_ films, or to identify alternative materials to achieve efficient and reliable devices.

## Graphical abstract

The paper reports the degradation of a critical tin oxide precursor, tetrakis(dimethylamido)tin(IV), into bis(dimethylamido)tin(II), during atomic layer deposition. While the growth and morphology remain unchanged, this degradation alters the film's electronic properties, reducing the performance of optoelectronic devices like perovskite solar cells, where tin oxide acts as an electron transport layer.

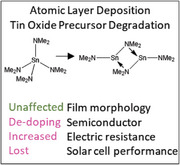

## 1 Introduction

Tin oxide (SnO_x_) thin films are gaining increasing attention from academia and industry due to their optical, electrical, and chemical properties.^[^ ^1^, ^2^, ^3^, ^4^, ^5^ ^]^ Its wide bandgap (3.5 eV), high electron mobility (260 cm^2^ V^−1^ s^−1^), and transparency to visible light make SnO_x_ an excellent semiconductor for a range of applications, including gas sensors, catalysts, transparent conductive electrodes, light‐emitting diodes, and photovoltaics.^[^ ^6^, ^7^ ^]^

**Figure 1:**
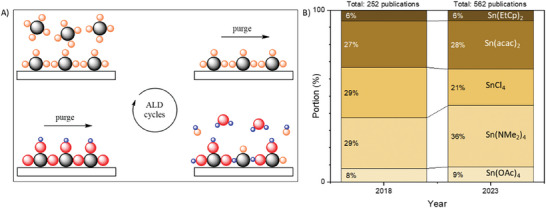
Atomic layer deposition (ALD). A) Schematic demonstrating layer‐by‐layer deposition of SnO_x_ film via ALD. B) Number of publications on “ALD”, “tin oxide” (OR “SnO_2_”, “SnO_x_)” with certain precursors (e.g., “TDMASn OR tetrakis(dimethylamino)tin”). The data were obtained from the Wiley Online Library.

Tin oxide thin films can be synthesized from premade SnO_x_ nanoparticles dispersed in solvents, as well as from tin precursors via chemical bath deposition, or atomic layer deposition (ALD). The ALD offers a set of advantages over other synthetic methods in light of its ability to form high‐quality films uniformly and conformally with atomic precision.^[^ ^4^, ^8^, ^9^, ^10^ ^]^ The self‐limiting nature of the ALD reaction ensures the deposition of only a monolayer of a material at a time, enabling precise control over film thickness (**Figure** **1**A).^[^ ^11^, ^12^, ^13^, ^14^, ^15^, ^16^, ^17^, ^18^, ^19^ ^]^ This is why ALD has become a leading method in fabricating tin oxide films.^[^ ^17^, ^20^, ^21^, ^22^, ^23^, ^24^ ^]^

Historically, tin chloride (SnCl_4_) has been the primary tin precursor for ALD of SnO_x_ films. However, both SnCl_4_ and its byproduct, HCl, are highly corrosive, prompting a gradual shift toward non‐halogenated sources. Tetrakis(dimethylamido)tin(IV) (TDMASn) emerged as a leading precursor choice for ALD of SnO_x_ films (Figure 1B) due to its volatility (with a vapor pressure of 0.04 Torr at 40 °C), which enables the synthesis of high‐quality films.^[^ ^18^, ^25^, ^26^, ^27^ ^]^ Several important studies have revealed that ALD parameters (such as temperature, carrier gas, and pulse times) impact the growth rate and properties of the ultimate film.^[^ ^26^, ^28^, ^29^, ^30^ ^]^

The TDMASn is conventionally considered a stable ALD precursor. However, it remains unknown if it indeed maintains its integrity under the conditions at which it is kept in the ALD system before and during deposition (80 °C or higher for a long period).^[^ ^11^, ^31^, ^32^, ^33^, ^34^, ^35^, ^36^, ^37^, ^38^ ^]^

**Figure 2:**
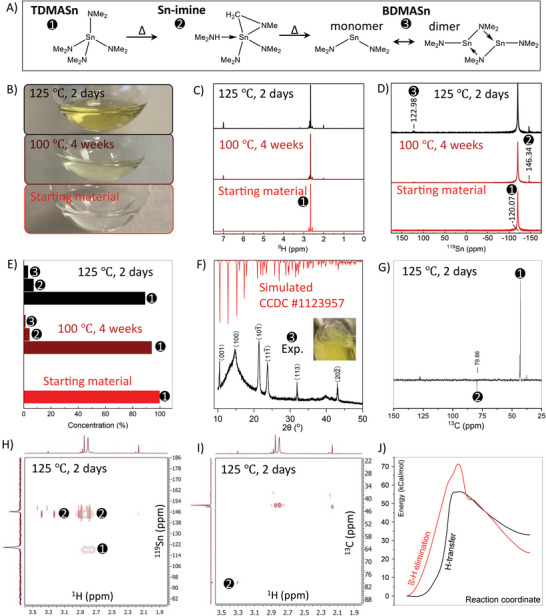
TDMASn and its degradation under heat treatment. A) Schematic showing degradation of TDMASn into BDMASn and its dimer through Sn‐imine. B) Pictures, C) ^1^H NMR (C_6_D_6_, 300 MHz), D) ^119^Sn NMR (C_6_D_6_, 186 MHz), and E) content of TDMASn before and after heat treatment. F) XRD of ground crystals formed in heat‐treated TDMASn. G) ^13^C NMR (C_6_D_6_, 126 MHz), (H) ^1^H‐^119^Sn HMBC NMR (C_6_D_6_), and I) ^1^H‐^13^C HSQC NMR (C_6_D_6_) of heat‐treated TDMASn. J) Comparison of DFT‐calculated degradation pathways of TDMASn.

Here we report a degradation of TDMASn into bis(dimethylamido)tin(II) (BDMASn) through the Sn‐imine complex (Sn─C─N metallacycle) at temperatures relevant to ALD conditions (**Figure** **2**A). We show that this degradation has a negligible influence on the growth kinetics and morphology of ALD‐SnO_x_ films, but it significantly alters their electronic properties. Such films show twice higher electrical resistance; and when employed in perovskite solar cells, they reduce power conversion efficiency significantly.

## 2 Results and Discussion

### 2.1 TDMASn (in)Stability

The TDMASn precursor, provided by Seastar Chemicals ULC in this study, appeared as a colorless liquid (Figure 2B). Its ^1^H and ^119^Sn nuclear magnetic resonance (NMR) spectra showed a singlet peak with satellites, corresponding to TDMASn, indicating the high purity of the precursor (Figure 2C–E).

To investigate the stability of TDMASn at elevated temperatures (i.e., conditions in which ALD precursor containers are kept), we heated it in an inert atmosphere. The precursor developed a yellowish color upon heat treatment (Figure 2B), and showed five additional peaks in its ^1^H NMR spectrum (Figure 2C), which we attribute to Sn‐imine complex (structure **2** in Figure 2A), as discussed below. Prolonged storage of TDMASn heat‐treated at 125 °C for 2 days led to the formation of transparent crystals (Figure 2F, inset), which we isolated and identified to be bis(dimethylamino)‐bis(µ‐dimethylamido)‐di‐tin(II) (BDMASn‐dimer) via X‐ray diffraction analysis (XRD, Figure 2F).^[^ ^39^ ^]^

The ^119^Sn NMR spectra of heat‐treated TDMASn showed resonance peaks corresponding to TDMASn, BDMASn‐dimer, and intermediate Sn‐imine complex **2** (Figure 2D).^[^ ^40^ ^]^ The ^13^C‐DEPT NMR spectrum (DEPT stands for distortionless enhancement by polarization transfer) exhibited a negative intensity peak (79.66 δ) corresponding to the ─CH_2_ carbene group, indicating the presence of the imine group in compound **2** (Figure 2G).

To elucidate the structure of compound **2** in heat‐treated TDMASn (125 °C for 2 days), we performed ^1^H‐^119^Sn Heteronuclear Multiple Bond Correlation (HMBC) and ^1^H‐^13^C Heteronuclear Single Quantum Coherence (HSQC) NMR spectroscopy (Figure 2H,I). The (^1^H,^119^Sn) (2.80 δ, −120.07 δ) crosspeak shown in Figure 2H represents TDMASn. Singlets due to Sn‐NMe_2_H (2.17 δ), Sn(NMe_2_)_2_ (2.86 δ), Sn‐NMeCH_2_ (2.88 δ), and Sn‐NMeCH_2_ (3.31 δ) are correlated to the same tin resonance −146.34 δ of compound **2** (Figure 2H). The (^1^H,^13^C) (3.31 δ, −79.66 δ) crosspeak shown in Figure 2G indicates the presence of ─CH_2_ carbene group in compound **2**. Thus, compound **2** was determined to be Sn‐imine (or Sn─C─N metallacycle complex), as shown in Figure 2A, containing the functional groups noted above. A similar structure was reported to form from tetrakis(dimethylamino)titanium(IV) upon heat treatment.^[^ ^41^ ^]^

To probe the feasibility of TDMASn degradation into BDMASn through the Sn‐imine complex via proton (H^+^) transfer mechanism, we performed density functional theory (DFT) calculations and indeed found this route to be more thermodynamically preferred (≈55 kCal mol^−1^ energy barrier) as compared to the alternative mechanism through beta‐hydride elimination (β‐H elimination, ≈70 kCal mol^−1^ energy barrier) (Figure 2J; Figure S1, Supporting Information). The β‐H elimination mechanism is energetically more demanding as it requires a square planar configuration with hydrogen positioned above the plane near the metal center. In addition, NMR spectra showed no trace of Sn─H interaction, which would form in the β‐H elimination route. Therefore, we conclude that the proton transfer is the dominant mechanism for the degradation of TDMASn to BDMASn, releasing dimethylamine and N‐methylmethanimine.

Subsequent ab initio molecular dynamics simulations showed that the dimethylamine and N‐methylmethanimine products do not remain coordinated with the BDMASn (Figure S2, Supporting Information). The calculations also showed that BDMASn molecules readily dimerize, forming BDMASn‐dimer in a trans configuration, which we also detected experimentally through XRD in solid crystals isolated from prolonged heat‐treated TDMASn (Figure S3, Supporting Information).

Figure 2E shows the quantification of heat‐treated TDMASn based on ^119^Sn NMR spectra in Figure 2D. As expected, increasing the temperature accelerated the degradation of TDMASn without altering the degradation products. For simplicity, we used TDMASn heat‐treated at 125 °C for 2 days, which contains ≈10% degradation products, in all subsequent studies: this sample will be referred to as HT‐TDMASn hereafter.

### 2.2 Impact of TDMASn Degradation on ALD‐SnO_x_ Growth and Composition

**Figure 3:**
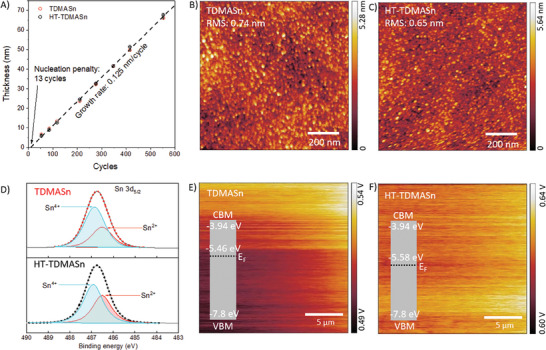
Impact of TDMASn degradation on properties of ALD‐SnO_x_ films. A) Thickness of ALD‐SnO_x_ films as a function of ALD cycles (0.5 s precursor pulse time). B,C) AFM topography, D) XPS spectra, and E,F) KPFM surface potential maps of 30 nm thick ALD‐SnO_x_ films. CBM and VBM in panels E and F stand for conduction and valence band maxima, respectively.

Following these findings, we aimed to understand how TDMASn degradation affects the ALD growth rate of SnO_x_ films. We used TDMASn and HT‐TDMASn to make SnO_x_ films via ALD and measured their thickness as a function of ALD cycles (**Figure** **3**A). Both TDMASn and HT‐TDMASn showed similar film growth rates, quantified as a slope of the curve in Figure 3A. Similarly, both precursors showed no measurable difference in the nucleation penalty, quantified as a cross‐section of the growth curve in Figure 3A extrapolated to the *x*‐axis (i.e., the number of ALD cycles required to form the first layer). These observations held even for much shorter ALD precursor pulses (Figure S4, Supporting Information).

In addition, the SnO_x_ films obtained from TDMASn and HT‐TDMASn demonstrated closely resembling morphology. Atomic force microscopy (AFM) images suggested that each set of samples exhibited a smooth topography, with statistically insignificant differences in surface root mean square (RMS) roughness (Figure 3B,C; Figures S5–S7, Supporting Information). We hence conclude that TDMASn and its degradation products have similar reactivity to each other (as indicated by the identical growth rates) and to the substrate, silicon in this study (as evidenced by the identical nucleation penalties).

Tin's oxidation state is +4 in TDMASn(IV) and +2 in BDMASn(II); therefore, we posited that the presence of BDMASn in HT‐TDMASn would impact the Sn^2+^/Sn^4+^ ratio in the ultimate ALD‐SnO_x_ films. X‐ray photoemission spectroscopy (XPS) of the films indeed showed that the films made from HT‐TDMASn contained 41% Sn^2+^ (Figure 3D), corresponding to SnO_1.59_ brutto‐formula ([Sn(II)O]_0.41_[Sn(IV)O_2_]_0.59_). In contrast, the film made from TDMASn contained 33% Sn^2+^, corresponding to SnO_1.67_ brutto‐formula ([Sn(II)O]_0.33_[Sn(IV)O_2_]_0.67_). To understand this difference, we performed DFT calculations and found that TDMASn can form both Sn(II)O and Sn(IV)O_2_ oxides, with Sn(IV)O_2_ being the more favorable product, whereas BDMASn preferentially forms Sn(II)O (Figures S8–S13, Supporting Information). We therefore conclude that the increase in Sn^2+^ in ALD‐SnO_x_ films made of HT‐TDMASn is due to the presence of BDMASn(II).

The XPS O1s peak deconvolution (Figure S14, Supporting Information) indicated that the peak area ratio of adsorbed oxygen O_x_^−^ (O^−^ and O_2_^−^) to lattice oxygen (Sn^2+^‐O and Sn^4+^‐O) is higher for HT‐TDMASn than for TDMASn (0.51 vs 0.47). This suggests that the HT‐TDMASn film is more deficient in oxygen, i.e., contains a higher concentration of oxygen vacancies.^[^ ^42^ ^]^

### 2.3 Impact of TDMASn Degradation on ALD‐SnO_x_ Electronic Properties

The electronic properties of SnO_x_ depend on the oxidation state of Sn.^[^ ^11^ ^]^ The higher the proportion of Sn^4+^ in SnO_x_, the greater the density of mobile electrons, leading to an *n*‐type semiconductor with a shallow work function (WF, the energy required to remove an electron from a solid). We measured the WF of the films via kelvin probe force microscopy (KPFM) and found that HT‐TDMASn leads to ALD‐SnO_x_ films with a higher WF (5.58 eV) compared to that of TDMASn (5.46 eV) (Figure 3E,F). This indicates that the ALD‐SnO_x_ made from HT‐TDMASn is less *n‐*doped (or more intrinsic) than the film made from TDMASn. This has significant implications for thin film devices as we show below.

**Figure 4:**
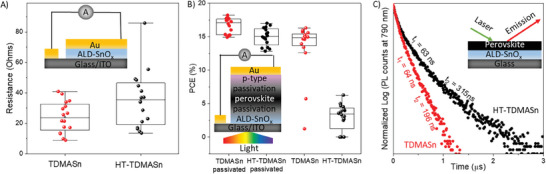
Impact of TDMASn degradation on devices employing ALD‐SnO_x_ films. A) Electrical resistance of ALD‐SnO_x_ films sandwiched between ITO and Au electrodes. B) Power conversion efficiency of perovskite solar cells with a structure of ITO/ALD‐SnO_x_/perovskite/Spiro/Au. Passivated devices had a structure of ITO/ALD‐SnO_x_/KCl‐passivation/perovskite/OAI‐passivation/Spiro/Au. C) Photoluminescence decay of perovskite film deposited on ALD‐SnO_x_ films.

To study the electrical properties of ALD‐SnO_x_, we sandwiched it between two electrodes – indium tin oxide (ITO) and gold (Au) – to create ITO/SnO_x_/Au devices (Figure S17, Supporting Information). We then measured current–voltage (*I–V*) characteristics from 16 independent devices and quantified the electrical resistance of SnO_x_ films as a slope of the *I–V* characteristics (**Figure** **4**A). SnO_x_ films made from HT‐TDMASn showed nearly twice the electrical resistance compared to those made from TDMASn, in agreement with the KPFM findings discussed above.

Tin oxide films are widely used in perovskite solar cells as an electron transporter layer due to their *n*‐type nature, and as an interconnection layer in tandem solar cells due to their high conductivity.^[^ ^43^, ^44^, ^45^, ^46^, ^47^ ^]^ To study the effect of Sn‐ALD precursor degradation on perovskite solar cell performance, we fabricated 16 perovskite solar cells in ITO/SnO_x_/FAPbI_3_ perovskite/spiro‐OMeTAD/Au (FA stands for formamidinium) architecture following protocols we recently reported, using only scalable techniques in ambient air.^[^ ^48^ ^]^ XRD and AFM of FAPbI_3_ perovskite films on ALD‐SnO_x_ films prepared from TDMASn and HT‐TDMASn showed no significant crystallinity and morphological differences (Figures S16 and S17, Supporting Information). However, solar cells employing TDMASn showed an average power conversion efficiency of 15%, while those with HT‐TDMASn showed only ≈4% efficiency (Figure 4B; Figure S18, Supporting Information).

We then passivated the surface of ALD‐SnO_x_ with KCl to eliminate surface defects,^[^ ^48^ ^]^ which increased the efficiency of solar cells employing TDMASn up to 18.2%. As for the non‐passivated counterparts, devices using HT‐TDMASn consistently showed lower efficiency, likely due to bulk defects in HT‐TDMASn‐derived ALD‐SnO_x_ film. Our control devices using blade‐coated SnO_x_ nanoparticles exceeded 20% efficiency, comparable to FAPbI_3_‐based perovskite solar cells fabricated via the all‐scalable blade‐coating method reported to date (Figure S19, Supporting Information). We attribute the significant decrease in power conversion efficiency to the high electrical resistance and defect density of ALD‐SnO_x_ films made by HT‐TDMASn, as discussed above, which led to high series resistance in the device (Figure S20 and S21, Supporting Information), as well as inefficient charge transfer at the SnO_x_/perovskite interface, as discussed next.

To study the efficiency of charge transfer from perovskite to SnO_x_ (the foundational mechanism of operation of solar cells), we prepared glass/SnO_x_/perovskite stacks. We tracked the photoluminescence (PL) decay of perovskite film (Figure 4C). An efficient charge transfer in this measurement should lead to fast PL decay. The PL decay was nearly twice as slow for ALD‐SnO_x_ made of HT‐TDMASn likely due to the defective nature of the latter, hence explaining the poor performance of corresponding solar cells discussed above. Water contact angle measurements showed that the films made by TDMASn are more wettable (Figure S22, Supporting Information), which typically leads to better perovskite coverage and hence better carrier extraction efficiency from perovskite to electron transporter layer.^[^ ^49^, ^50^, ^51^ ^]^

## 3 Conclusion

In conclusion, we reported a significant degradation of a critical ALD precursor, TDMASn, into BDMASn and Sn‐imine complex under heat stress. We found that such degradation does not affect the growth kinetics and morphology of SnO_x_ films, yet it is detrimental to electronic properties, such as charge transport and transfer efficiency. As a result, perovskite solar cells employing such films exhibited a significant loss in power conversion efficiency. Future studies should focus on developing strategies to prevent these degradation mechanisms and identifying the primary factors that cause impurities, aside from heating the precursor.

## Conflict of Interest

The authors declare no competing financial interest.

## Author Contributions

S.Q., A.A., D.F., and J.A.M. contributed equally to this work. S.Q., D.F., and I.P. performed conceptualization. S.Q., A.A., D.F., J.A.M., C.C., D.Y., V.Y., I.T.C., and I.P. performed the investigation. M.I.S. provided resources. S.Q. wrote the original draft. M.I.S., A.A., D.F., J.A.M., IT.C., and I.P. wrote, reviewed, and edited the final manuscript. M.I.S. performed supervision.

## Supporting information



Supporting Information

## Data Availability

The data that support the findings of this study are available in the supplementary material of this article.
