# Supplementary material for: Impact of Tetrakis(dimethylamido)tin(IV) Degradation on Atomic Layer Deposition of Tin Oxide Films and Perovskite Solar Cells
Source: Small. 2024 Nov 6;21(1):2404966. doi: 10.1002/smll.202404966 (PMC11707587; doi:10.1002/smll.202404966)
Supplement: Supplementary file 1 — Supporting Information [file SMLL-21-2404966-s001.pdf]

NANO · MICRO
small

Supporting Information

for *Small*, DOI 10.1002/smll.202404966

Impact of Tetrakis(dimethylamido)tin(IV) Degradation on Atomic Layer Deposition of Tin Oxide Films and Perovskite Solar Cells

Shuang Qiu, Augusto Amaro, Diana Fabulyak, Julien Appleby-Millette, Cassidy Conover, Dongyang Zhang, Vishal Yeddu, I Teng Cheong, Irina Paci and Makhsud I. Saidaminov**

Supplementary Information

for

Impact of Tetrakis(dimethylamido)tin(IV) Degradation on Atomic Layer Deposition of Tin Oxide Films and Perovskite Solar Cells

Shuang Qiu[1&], Augusto Amaro[1&], Diana Fabulyak[2&], Julien Appleby-Millette[1&], Cassidy Conover[2], Dongyang Zhang[1], Vishal Yeddu[1], I Teng Cheong[1], Irina Paci[1,*], Makhsud I. Saidaminov[1,3,4*]

[1]Department of Chemistry, [3]Department of Electrical and Computer Engineering, [4]Center for Advanced Materials and Related Technologies (CAMTEC), University of Victoria, Victoria, British Columbia V8P 5C2, Canada

[2]Seastar Chemicals ULC, 2061 Henry Avenue West, Sidney, BC, Canada V8L 5Z6, Canada

[&]These authors contributed equally to this work

*E-mail: ipaci@uvic.ca; msaidaminov@uvic.ca

Experimental

1. TDMASn degradation forming BDMASn-dimer

Colorless liquid TDMASn precursor was loaded into a glass ampoule and sealed under vacuum. This sample was heated at 125 °C for 48 hours without exposure to light. After this heating process, the sample turned yellow and NMR analysis confirmed the formation of side products. Solid suitable for an X-ray diffraction analysis crashed out of this mixture at room temperature after 2 weeks.

2. Fabrication of ALD SnO_x thin films

A thermal atomic layer deposition reactor (Veeco Savannah S100 ALD) was used to deposit the samples. TDMASn (or HT-TDMASn) and H_2O were used as the precursor and the co-reactant, respectively, to fabricate the SnO_x thin films. A high-purity (99.99%) N_2 source was used as the carrier gas. For the TDMASn precursor, a TDMASn precursor provided by Seastar Chemicals ULC was used. For the HT-TDMASn precursor, the same material underwent a heating process to intentionally add ~10% impurity. The deposition temperature was maintained at 80°C, and the precursors' pulse and purge times were set to be 0.5 s/30 s/0.015 s/60 s for TDMASn/purge/H_2O/purge, respectively. During the time ALD is on standby, the TDMASn cylinder is kept at 50 °C maintaining it volatile for deposition.

3. Characterization of TDMASn precursor and SnO_x films

Depositions were conducted on Si (100) substrates with a native SiO_2 layer. The Si wafers were cleaned with isopropanol (IPA) and wipe cleaning method, followed by 30 minutes of UV-ozone cleaning right before ALD deposition.

To analyze the thickness and growth rate the ellipsometry method with Alpha-SE, J. A. Woollam Co equipment was used. A modified Cauchy model based on the library model was used to fit the obtained SnO_x ellipsometry data. The morphological structure of the ALD SnO_x films was obtained using a Keysight 5500 Scanning Probe Microscope, and the topography and roughness (RMS) data were processed using Gwyddion 2.62 software. Kelvin Probe Force Microscopy (KPFM) images were obtained using the MFP-3D SPM equipment by Asylum Research to analyze the surface potential map and calculate the work functions of both fresh and aged ALD-SnO_x deposited over ITO substrates. For these calculations, an 80 nm gold sample was used as a reference. Nuclear Magnetic Resonance (NMR) analysis was carried out with AV III 300 to deduct the intramolecular behavior of the TDMASn-aged precursor. The SnO_x deposited on Si(100) substrates with fresh and aged TDMASn precursors was analyzed by X-ray photoelectron spectroscopy (XPS) using Thermofisher Scientific K-Alphaand, and the data were analyzed by XPS Peak 41. The effect of the impurity in the SnO_x film was

analyzed by using it as charge carrier transport for electron transport layers (ETL). Photoluminescence spectroscopy (PL) was conducted employing a confocal Raman microscope provided by Renishaw with the excitation at 633 nm. Time-resolved photoluminescence (TRPL) spectra were obtained employing an Edinburgh Instruments OB920 single photon counter equipped with a pulsed laser diode, and the signal detection was performed at 790 nm with a 16 nm bandwidth monochromator. To understand the impact of the impurity on the conductivity of the ALD-SnO_x, devices with the glass/ITO/ALD-SnO_x/Au structure were fabricated and measured using the 2-point probe method and a Keithley 2450 source meter.

4. Computational Method

The degradation pathway was also studied using DFT calculations using ORCA 5.0.3[1] quantum computing software using the PBE0 hybrid functional with the valence triple zeta polarization basis set (def2-TZVP) and D4 dispersion model.[2] A tight gird (defgrid3) was chosen to avoid rotational bias during the calculations and an aqueous implicit solvent was implemented using a continuum solvation method (CPCM SMD). The SMD method forced the use of numerical frequency calculation to confirm transition state saddle points. Loose climbing-image nudged elastic band calculations (Loose NEB-CI) were run to evaluate the competing beta-hydride elimination the hydrogen transfer mechanisms and the formation and inter-conversion of the dimer. Ab-initio Molecular Dynamics (AIMD) simulations were run on SIESTA 4.15[3] using the PBE GGA function to evaluate the stability of the coordinated reduction product. Further NEB calculations were evaluated over the aqueous oxidation pathway steps of BDMASn and TDMASn to both the SnO and SnO_2 products and combined into continuous pathways based on the uncoordinated geometries between each step.

5. Solar cell device fabrication

Perovskite solution *preparation*: The $FAPbI_3$ perovskite ink (1.0 M) was prepared by dissolving PbI_2, FAI and CdI_2 in 2-Methoxyethanol (2-ME). MACl (30 mol%), L-α-phosphatidylcholine (0.05 wt%) and NMP (5 vol%) were added to the precursor before use to improve the uniformity and coverage.

Solar cells with ALD-SnO_x: Glass substrate with ITO patterns was cleaned with detergent, deionized water, acetone, and isopropanol for 20 mins in an ultrasonic bath, respectively, followed by UV-ozone treatment for 30 mins. Then ALD-SnO_x was deposited as described in section 2 above. 30 μL perovskite ink was deposited on the annealed KCl/SnO_2 layer by a one step blade coating with a nitrogen knife assistant at room temperature. The gap height between the substrates and applicator was set as 200 μm at a speed of 10 mm/s. The final perovskite films were annealed at 150 °C for 30 mins after coating. The hole transporting material's (HTM)

solution was prepared by dissolving 100 mg of Spiro-OMeTAD (Xi'an Polymer Light Technology Corp), then, 23 μL of Li-TFSI (Sigma–Aldrich) solution (540 mg in 1 mL ACN), 39 μL of TBP (tert-butylpyridine) (Sigma–Aldrich) and 10 μL of Co(III) TFSI salt (GreatCell Solar, FK209) (376 mg in 1 mL ACN) were added to this solution. 30 μL of the as prepared HTM solution was blade coated at a rate of 30 mm/s with a gap height of 150 μm. Finally, gold electrodes with a thickness of 80 nm and an active area of 0.049 cm^2 were applied using EvoVac thermal evaporation system by Angstrom Engineering Inc., under a vacuum of 10^{-6} Torr.

Solar cells with SnO_x nanoparticles: 30 μL SnO_2 and water mixed solution (1:6, volume ratio) was deposited on the pre-cleaned glass/ITO substrates, as describe above, by blade coater (ZEHNTNER ZAA 2300 automatic film applicator) on a base heated to 80 °C hot plate at a speed of 10 mm/s, and annealed at 150 °C for 30 mins after the deposition.

Passivation: ITO/SnO_x substrates were treated with KCl solution (3 mg/mL in distilled water) before deposition of perovskite layer.[4] The n-Octylammonium iodide (OAI) post treatment consisted in coating 30 μL of OAI solution (2 mg/mL in isopropanol) on top of perovskite films after cooling.[5]

Device performance: The performance of devices were measured utilizing Newport Oriel sol-3A (class AAA) solar simulator under standard AM 1.5 conditions with an effective aperture area of 0.049 cm^2.

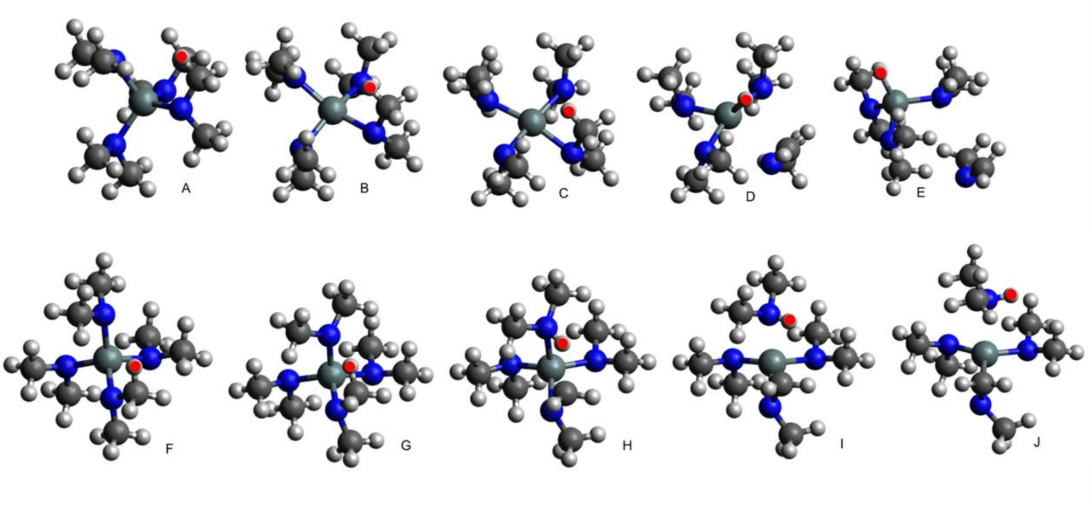

Figure S1. The NEB geometries along the Beta Hydride Elimination (A-E) and Hydrogen Transfer (F-J) pathways. The reactive hydrogen is highlighted with a red circle.

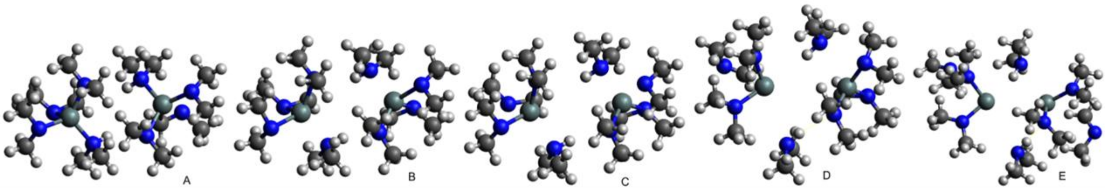

Figure S2. The AIMD trajectory of the BDMASn product with coordinated side products from the reduction reaction (A-E).

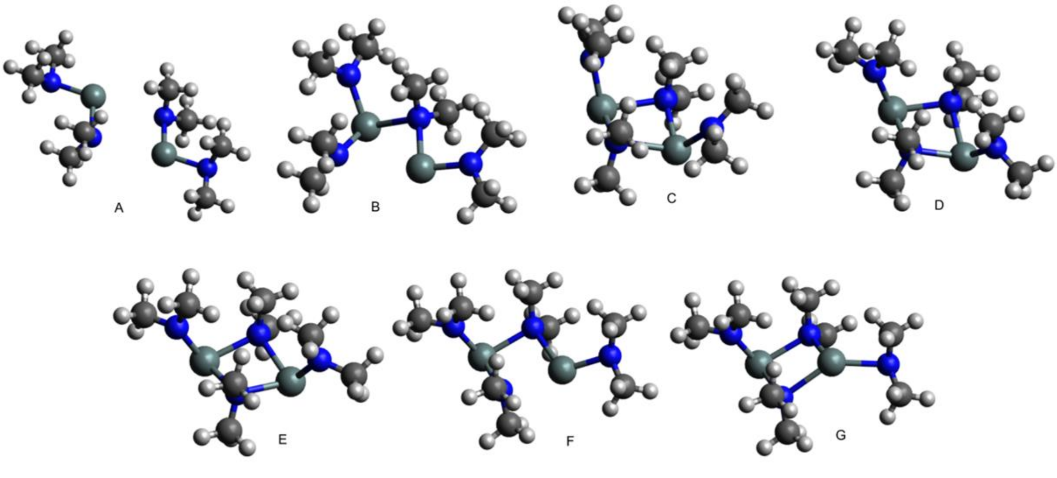

Figure S3. The minimization pathway for the formation of the BDMASn dimer (A-D) and the minimum energy pathway for the *cis* to *trans* dimer interconversion.

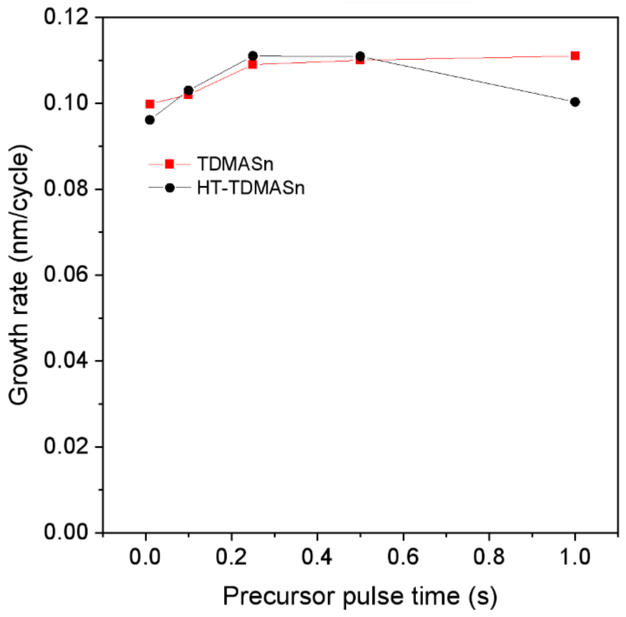

Figure S4. ALD-SnO_x film growth rate comparison with different pulse times.

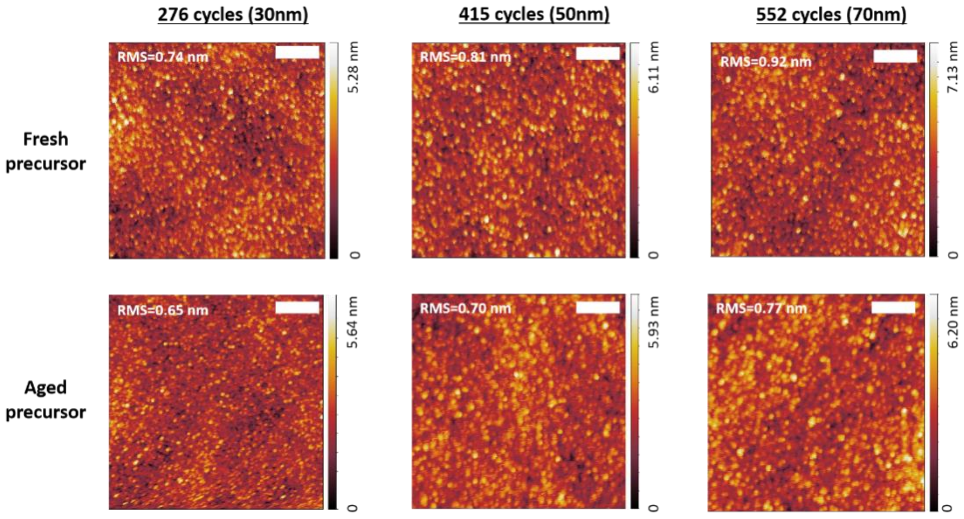

Figure S5. AFM topography images of ALD-SnO_x films. The scale bar indicates the length of 200 nm.

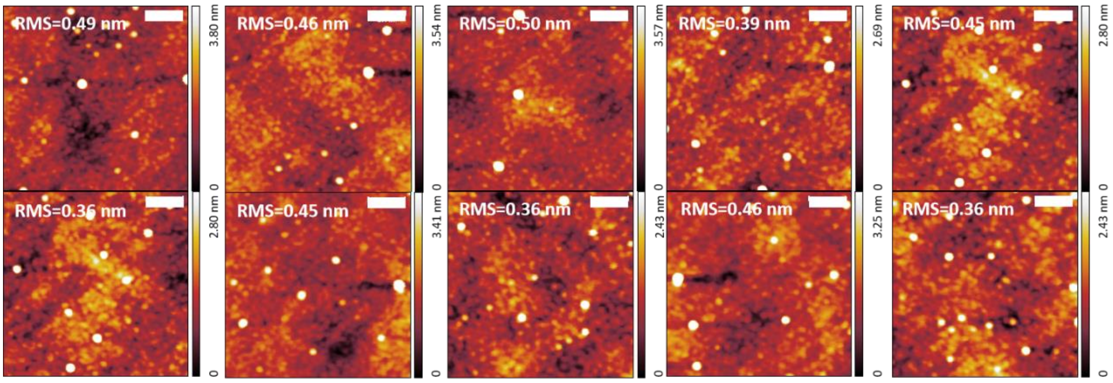

Figure S6. AFM topography of ten positions on a 20 nm ALD-SnO_x on Si(100) made with TDMASn. The RMS roughness uncertainty is ± 0.14 nm. The scale bar indicates the length of 200 nm.

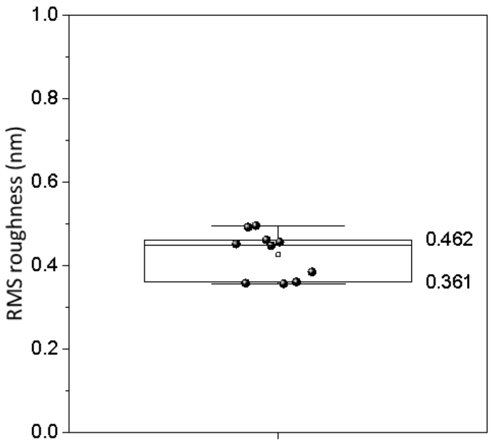

Figure S7. Statistical analysis of RMS roughness distribution for the 20 nm ALD-SnO_x.

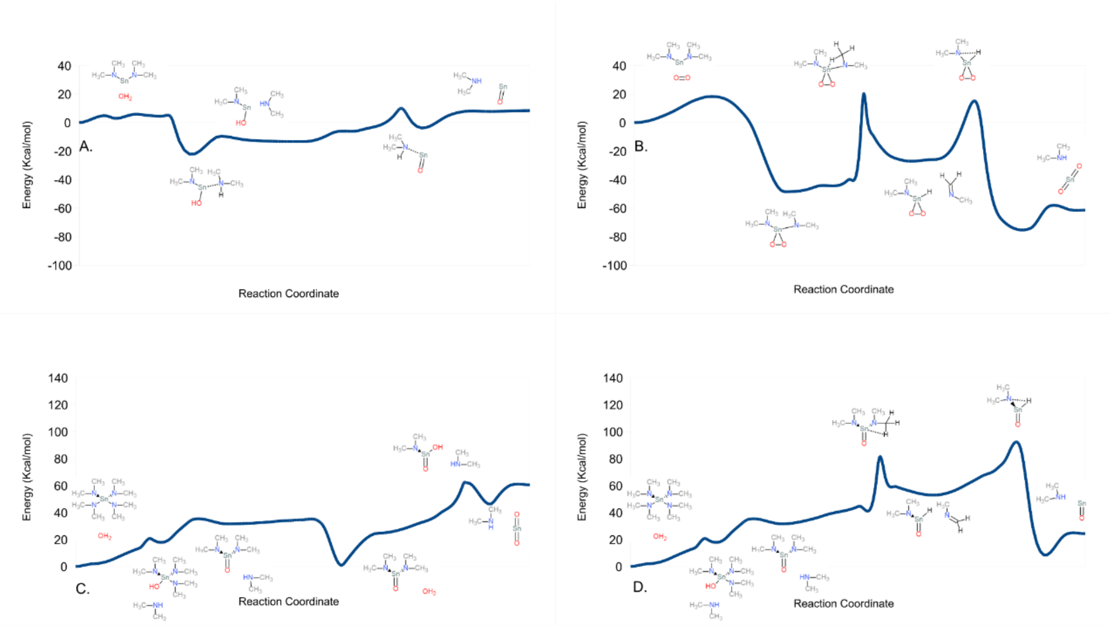

Figure S8. The DFT calculated oxidation pathways of BDMASn (A and B) and TDMASn (C and D) to both SnO (B and C) and SnO_2 (A and D). TDMASn is more likely to oxidize to the SnO_2 product and the BDMASn is more likely to oxidize to the SnO product. The two less favored pathways involve β-H elimination steps which have a configurational energy penalty.

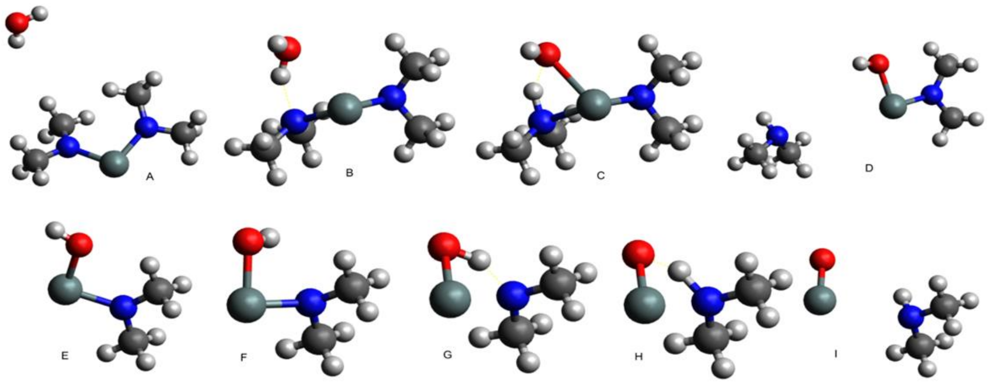

Figure S9. The oxidation pathway from BDMASn with H_2O to SnO (A-D, E-I).

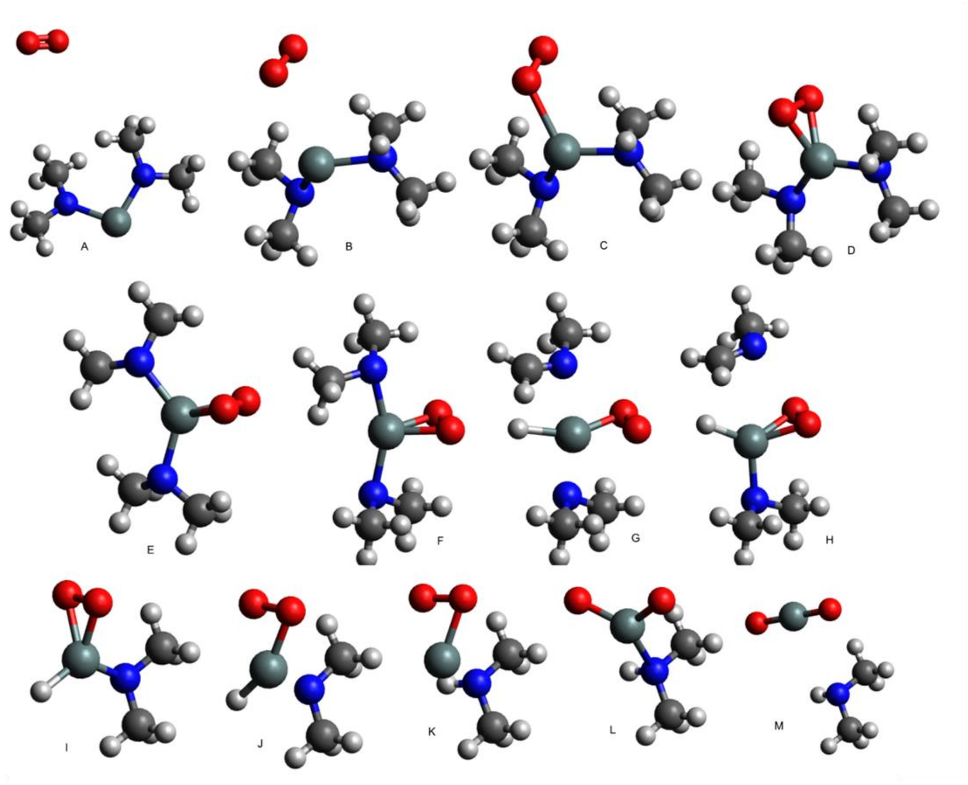

Figure S10. The oxidation pathway from BDMASn with O_2 to SnO_2 through a beta hydride elimination. (A-D,E-H,I-M).

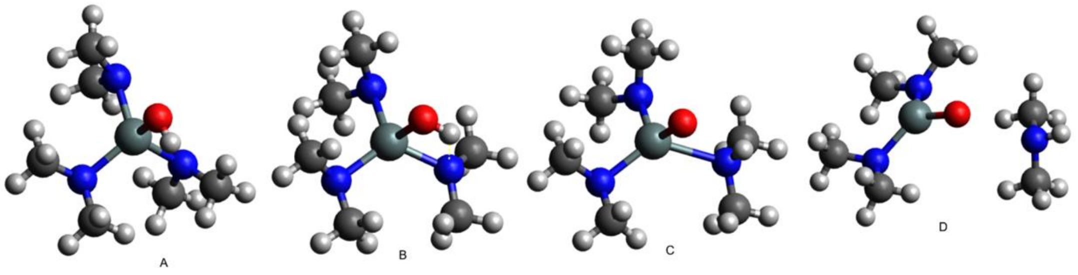

Figure S11. The initial oxidation pathway for TDMASn with H_2O (A-D).

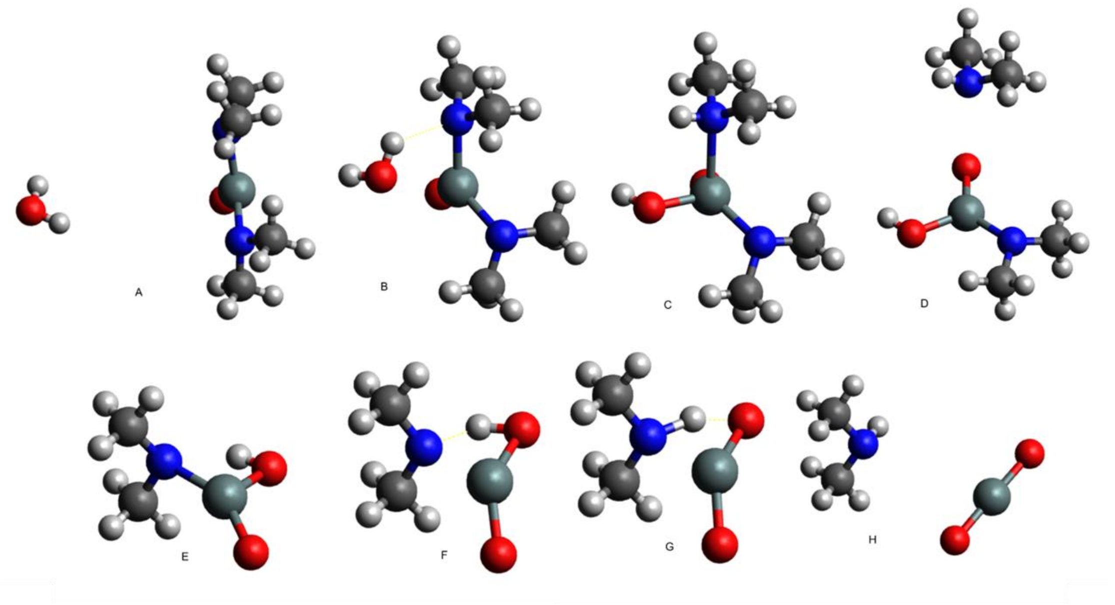

Figure S12. The oxidation pathway from BDMASnO with H_2O to SnO_2 (A-D,E-H).

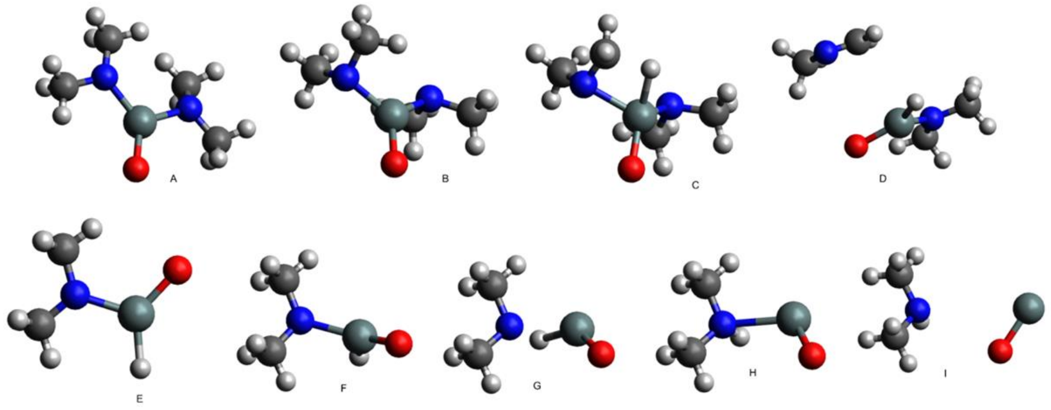

Figure S13. The oxidation pathway from BDMASnO to SnO through beta hydride elimination (A-D,E-I).

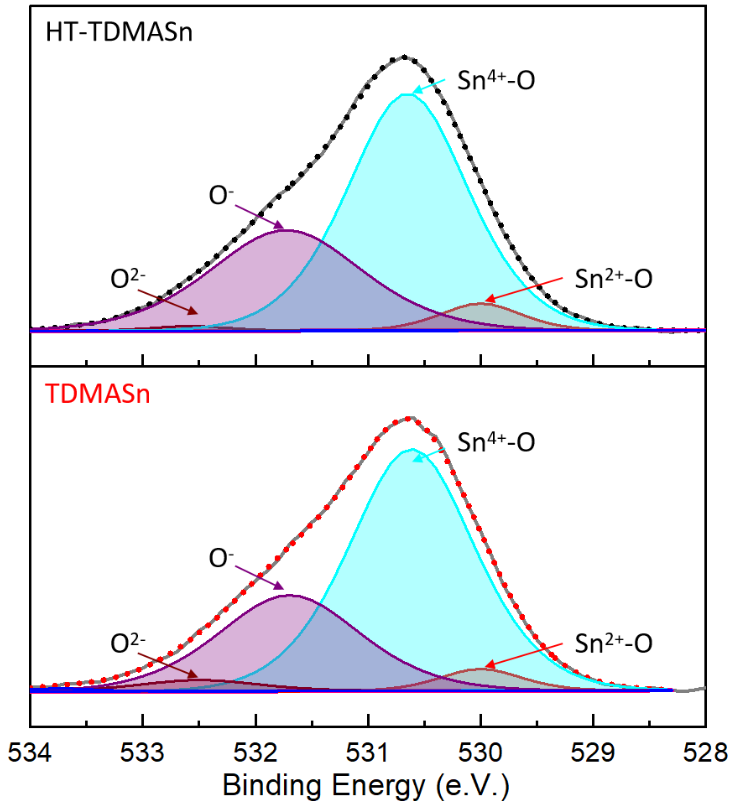

Figure S14. XPS O1s spectra of ALD-SnO_x film (made with TDMASn or HT-TDMASn) coated on Si(100) wafer.

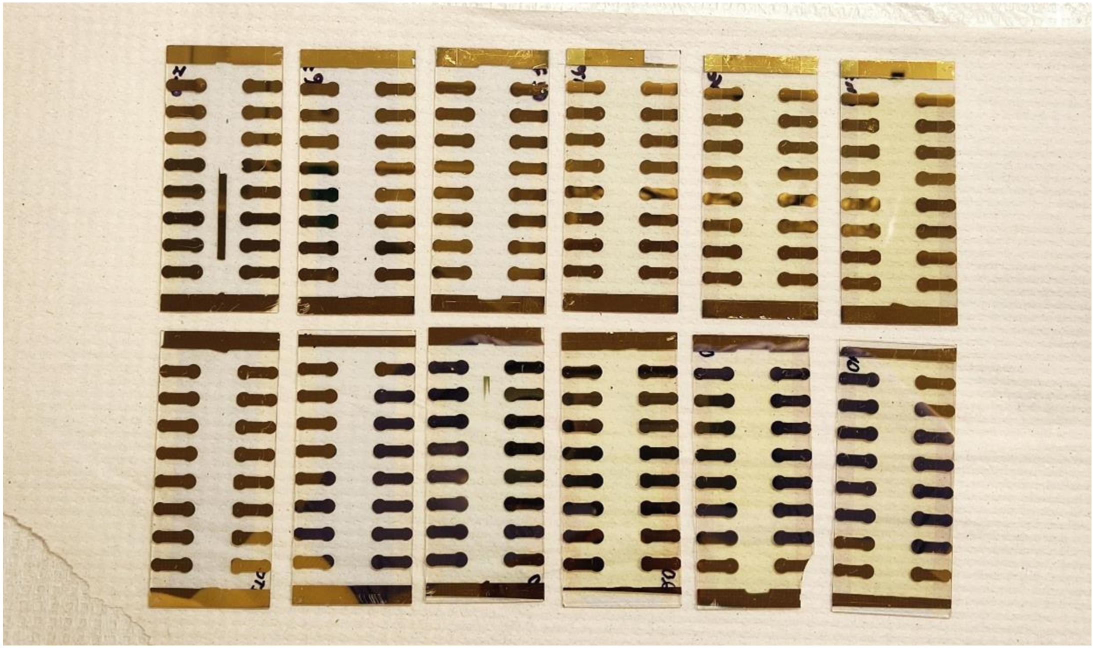

Figure S15. ITO/SnO_x/Au devices for current-voltage (I-V) characteristics.

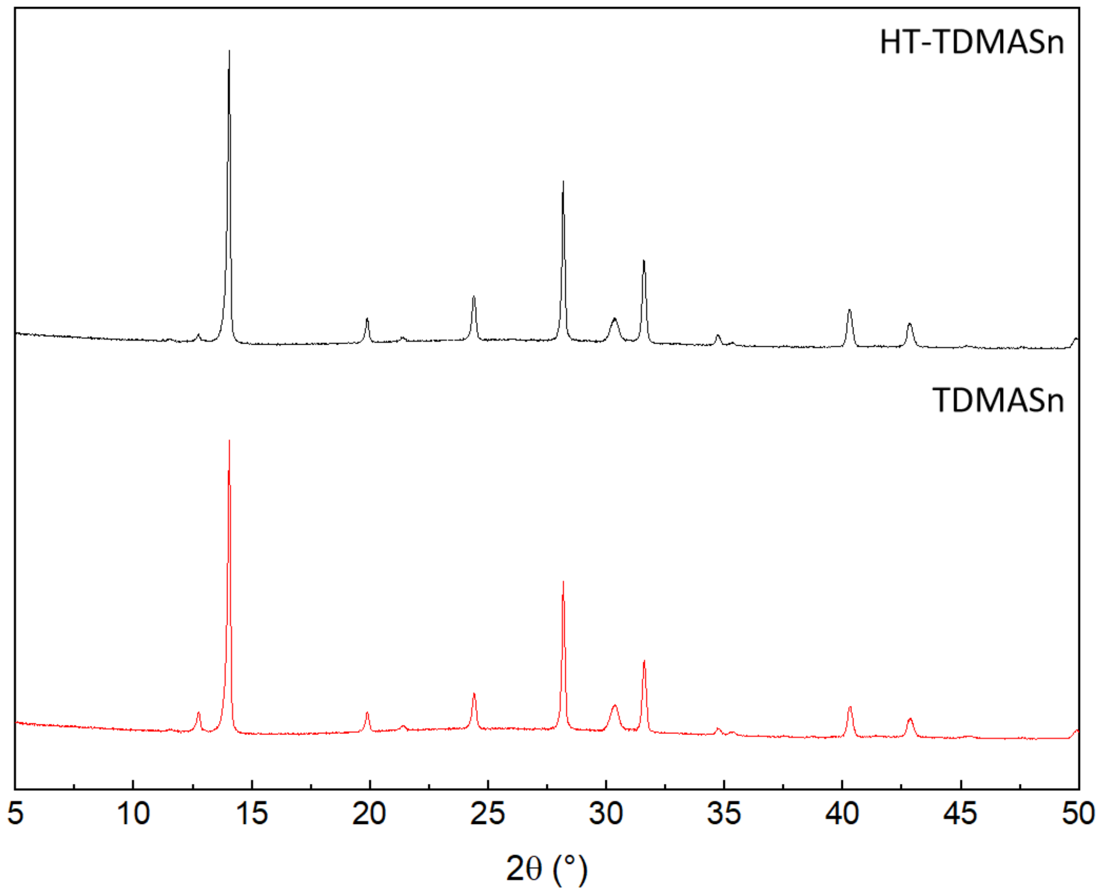

Figure S16. X-Ray diffraction spectra of $FAPbI_3$ perovskite films on ALD-SnO_x films made from TDMASn (bottom panel) and HT-TDMASn (top panel).

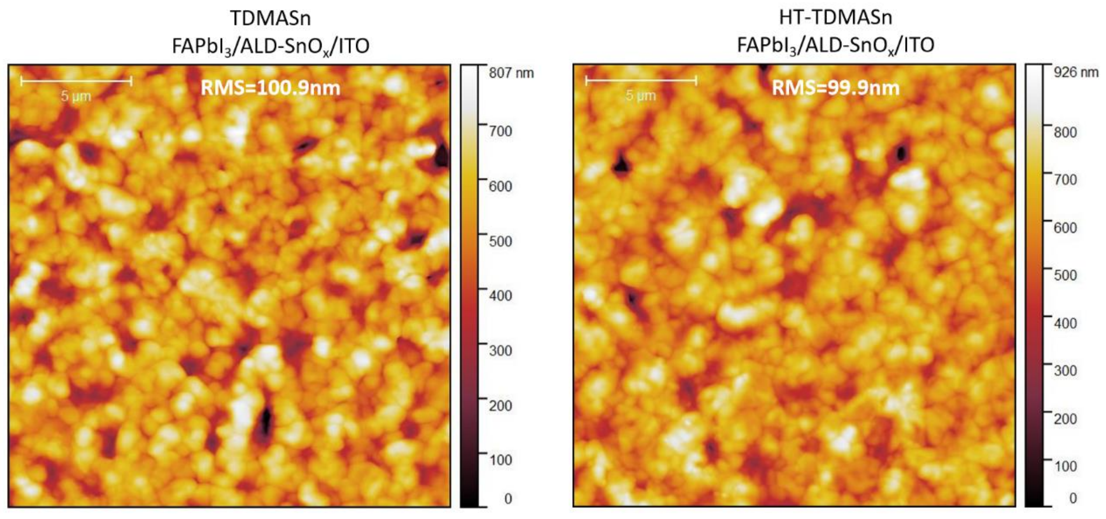

Figure S17. AFM image of the $FAPbI_3$ film topography on ALD-SnO_x film.

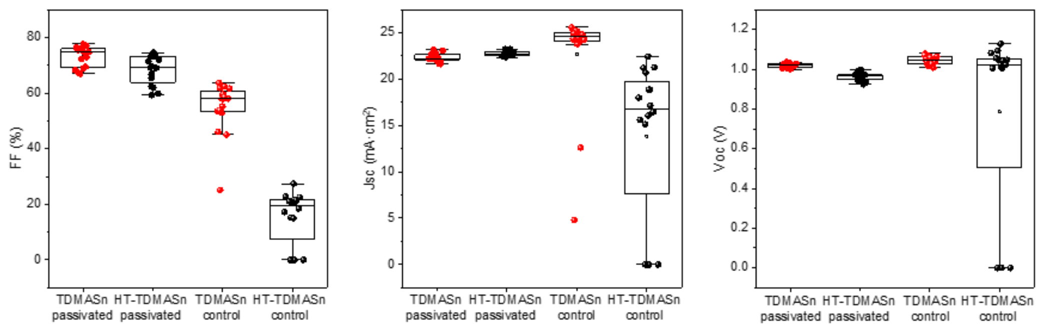

Figure S18. Photovoltaic parameters of solar cells using using ALD-SnO_x and blade-coated $FAPbI_3$ perovskite in ambient air. V_{OC} stands for open-circuit voltage, J_{SC} stands for short-circuit current, FF stands for fill factor.

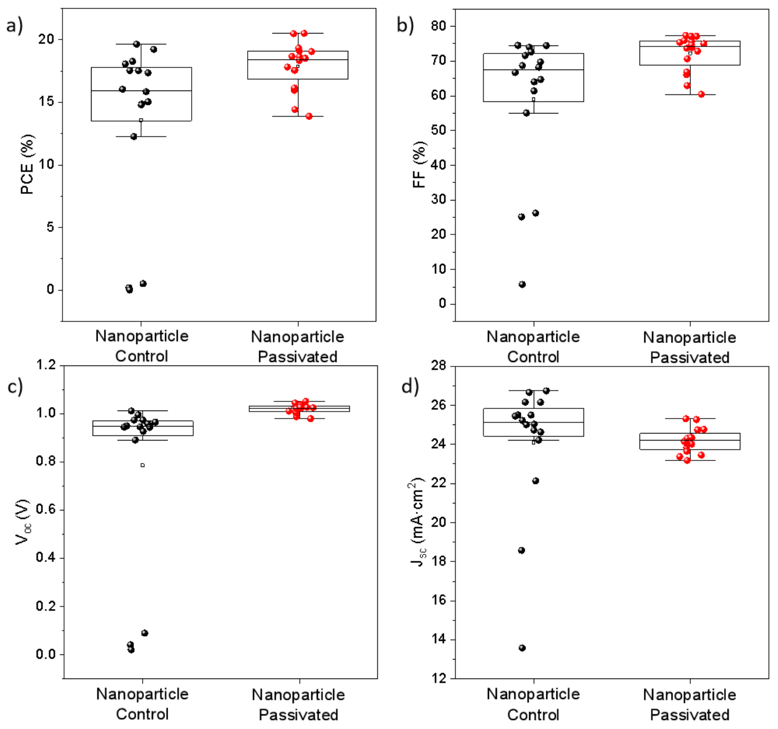

Figure S19. Photovoltaic parameters of solar cells using SnO_x nanoparticles and blade-coated $FAPbI_3$ perovskite in ambient air. The devices had a structure of ITO/SnO_x nanoparticles/KCl-passivation/perovskite/OAI-passivation/Spiro/Au. PCE stands for power conversion efficiency, V_{OC} stands for open-circuit voltage, J_{SC} stands for short-circuit current, FF stands for fill factor.

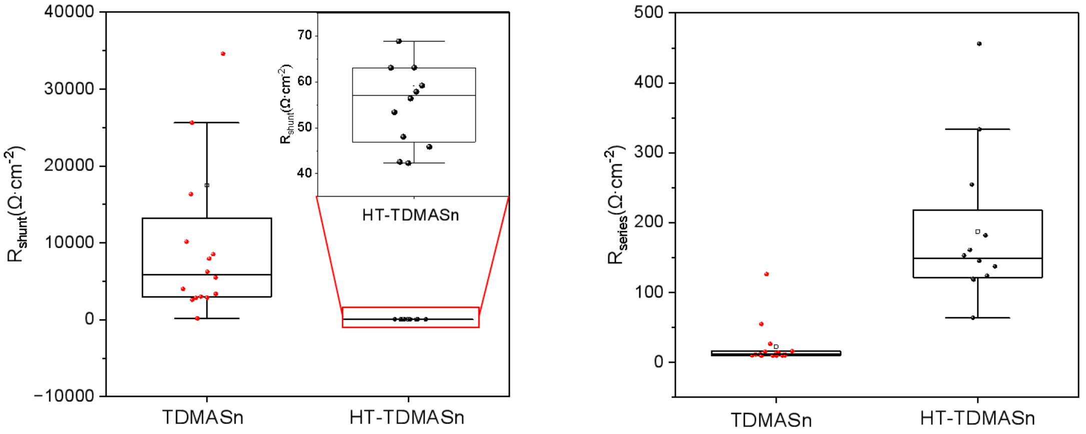

Figure S20. Parasitic resistance of perovskite solar cells without passivation. R_{shunt} stands for shunt resistance, R_{series} stands for series resistance.

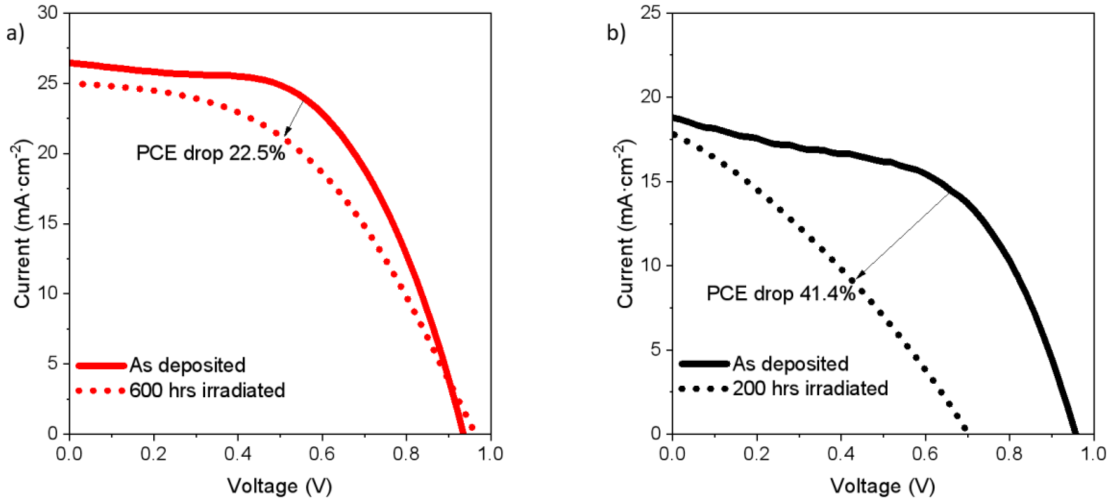

Figure S21. J-V curve comparison of perovskite solar cells without passivation as prepared (solid lines) and aged under light irradiation (dotted lines). The device from TDMASn has been aged for ~600 hrs; while the device from HT-TDMASn has been aged for ~200 hrs.

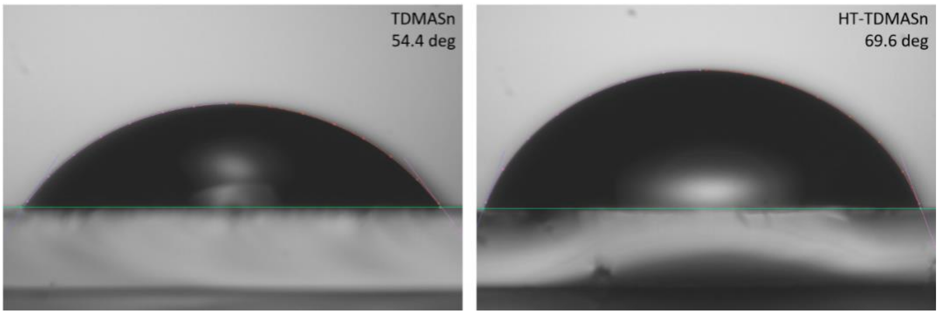

Figure S22. Contact angle of water droplet on ALD-SnOx films made from TDMASn or HT-TDMASn on ITO substrates.

Reference

[1] Neese F., Wennmohs, F., Becker, U., Riplinger, C., J. Chem. Phys., https://doi.org/10.1063/5.0004608.

[2] Caldeweyher E., Bannwarth C., Grimme S., J. Chem. Phys., https://doi.org/10.1063/1.4993215.

[3] Soler J. M., Artacho E., Gale J. D., García A., Junquera J., Ordejón P., Sánchez-Portal D., J. Phys.: Condens. Matter., https://doi.org/10.1088/0953-8984/14/11/302.

[4] P. Zhu, S. Gu, X. Luo, Y. Gao, S. Li, J. Zhu, H. Tan, Adv. Energy Mater., https://doi.org/10.1002/aenm.201903083.

[5] H. Kim, S. Lee, D.Y. Lee, M.J. Paik, H. Na, J. Lee, S. II Seok, Adv. Energy Mater., https://doi.org/10.1002/aenm.201902740.
